# Conditions Affected by Storms

**Published:** 1896-02

**Authors:** C. Carleton Smith

**Affiliations:** Philadelphia, Pa.


### CONDITIONS AFFECTED BY STORMS.

C. Carleton Smith, M. D., Philadelphia, Pa.

Replying to the inquiry, what remedies have indications for ailments aggravated by storms, I may say that Gelsemium is one of the most useful remedies in conditions affected by *storms*. Also, as you well know, *Rhododendron*. But in the *Repertory of Modalities* there are no indications given of any drug which has "amelioration when storm breaks."

Restlessness, with attacks of anxiety, *especially* during a thunder-storm calls for *Nat-carb.* and *Phosphorus.* Nat-carb. also for menstrual troubles *before* thunder-storm.

Twitching of eyeballs and eyelids, with trembling of hands and legs and debility at every approaching thunder-storm calls for *Agaricus.*

Many ailments worse *before* and *during* thunder-storm points to *Petroleum.*

Feeling of restlessness in his blood *days before* a thunder-storm indicates *Psorinum.*

*Debility,* with sleepiness during storm, is Silicea.

Cough worse before a thunder-storm is Phosphor.

Rhododendron generally has *acute pains,* sharp and darting, very violent; also *tearing* before and during a thunder-storm. But it must not be repeated oftener than *once* in *seven days.* Grand remedy for gouty people whom storms affect.

In female patients affected by storms study always Pulsatillla first.

Persons whose spines have been injured and always suffer acutely *before* a storm breaks, give Hypericum. This latter remedy, by the way, is a most wonderful drug when there has occurred a nerve lesion. I have cured a lady who had been suffering from such a condition in a single spot on the inner side of the left thigh for many years. She had consulted numerous doctors without any relief. One prescription of Hypericum cured her entirely.

